# Case Report: Remarkable response to savolitinib plus chemotherapy and bevacizumab in second-line treatment of MET amplified colon cancer with liver metastasis

**DOI:** 10.3389/fonc.2026.1861675

**Published:** 2026-09-01

**Authors:** Ying Huang, Pei Shu, Shiyuan Gu, Feng Wen, Xin Wang

**Affiliations:** 1 Division of Abdominal Tumor Multimodality Treatment, Cancer Center, West China Hospital of Sichuan University, Chengdu, China; 2 Department of Radiation Oncology, Cancer Center, West China Hospital of Sichuan University, Chengdu, China

**Keywords:** bevacizumab, chemotherapy, combination therapy, MET amplification, metastatic colorectal cancer, savolitinib

## Abstract

**Background:**

Metastatic colorectal cancer (mCRC) is associated with poor prognosis and low survival rates, with acquired resistance limiting the durability of standard therapies. Mesenchymal-epithelial transition factor (MET) amplification is a rare but potentially actionable molecular alteration that has been implicated in treatment resistance, while clinical evidence supporting MET-directed combination therapy in mCRC remains limited.

**Case presentation:**

We report a rare case of a young patient with KRAS/NRAS/BRAF wild-type descending colon cancer with liver metastases and *de novo* MET amplification detected in the pretreatment primary tumor. After achieving stable disease with second-line FOLFIRI plus bevacizumab, savolitinib, a highly selective MET tyrosine kinase inhibitor (TKI), was added to the ongoing regimen. The patient subsequently achieved marked radiologic and pathological regression during combination treatment, suggesting a possible additive contribution of MET inhibition.

**Conclusion:**

Our findings suggest that the addition of savolitinib to standard chemotherapy and a VEGF inhibitor may have contributed additional antitumor activity in this patient with MET-amplified mCRC. However, the independent contribution of savolitinib cannot be determined, and this observation should be regarded as hypothesis-generating and warrants validation in prospective studies.

## Background

Colorectal cancer is the third most commonly diagnosed cancer worldwide, and metastatic colorectal cancer (mCRC) continues to carry a dismal prognosis, with a 5-year survival rate below 20% (1). Although the integration of VEGF- or EGFR-targeted agents with fluoropyrimidine-based chemotherapy plus oxaliplatin or irinotecan has improved survival, acquired resistance is nearly universal, leaving limited options for subsequent treatment (2–4).

In mCRC, *de novo* MET amplification has been identified as a rare but clinically relevant molecular alteration, with an incidence of less than 3%. MET amplification has been implicated in therapeutic resistance, particularly resistance to EGFR-targeted therapy, whereas aberrant HGF/MET signaling more broadly has been associated with chemotherapy resistance, tumor invasion, and metastatic progression (5–9). Increased c-Met expression and HGF/MET signaling have also been implicated in the biology of colorectal liver metastasis (10). A preclinical study has shown that c-Met gene knockdown significantly inhibited the proliferative activity and invasive capacity of colorectal cancer liver metastasis organoids, providing a biological rationale for exploring MET-directed therapy in selected patients (10).

Savolitinib is an oral, selective MET tyrosine kinase inhibitor with demonstrated clinical activity in locally advanced or metastatic non-small cell lung cancer harboring MET exon 14 (METex14) skipping alterations (11, 12). In addition, savolitinib has demonstrated encouraging clinical activity in papillary renal cell carcinoma and gastric cancer (11). Nevertheless, clinical evidence supporting its use in mCRC remains scarce.

**Figure 1:**
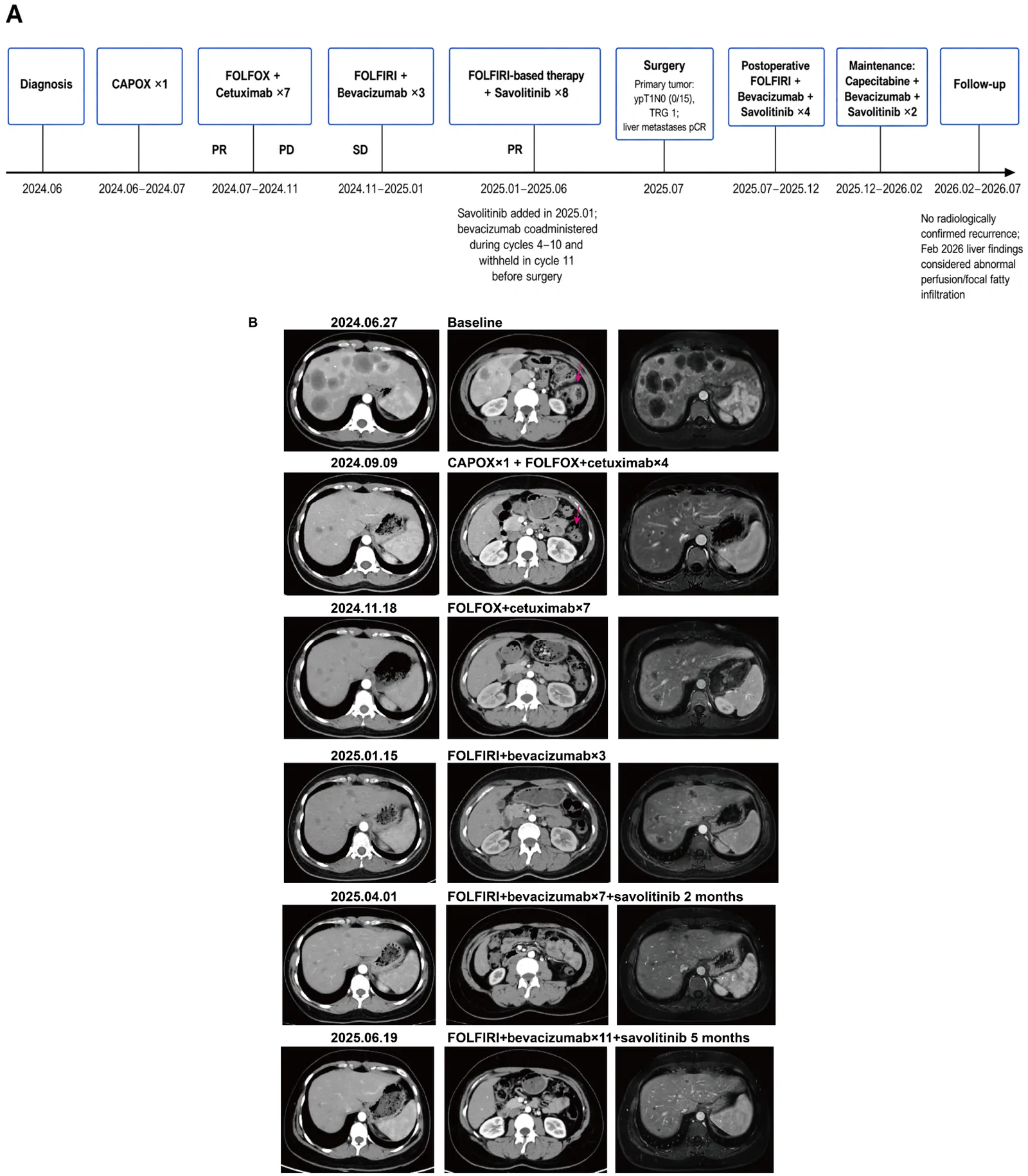
**(A)** Timeline of the whole treatment process for the patient. PR, partial response; PD, progressive disease; SD, stable disease; TRG, tumor regression grade. **(B)** Clinical images of the patient.

Here, we report a rare case of descending colon cancer with liver metastases and *de novo* MET amplification detected by next-generation sequencing in a pretreatment primary-tumor biopsy. The patient received standard chemotherapy combined separately with an EGFR inhibitor and with a VEGF inhibitor. During continued second-line FOLFIRI plus bevacizumab with the addition of savolitinib, the patient subsequently achieved a partial response, enabling successful conversion from unresectable to resectable disease. Postoperative pathological assessment demonstrated complete regression of the liver metastases, while the primary tumor showed a near-pathological complete response (near-pCR) (**Figure 1A**). However, because FOLFIRI and bevacizumab were continued after savolitinib initiation, the independent contribution of savolitinib could not be determined. This case should therefore be regarded as a hypothesis-generating observation suggesting a possible additive role for MET inhibition.

## Case presentation

In June 2024, a 32-year-old woman was admitted to West China Hospital,Sichuan university because of altered bowel habits and lower abdominal pain. She had no significant prior medical history. After admission, abdominal computed tomography (CT), abdominal magnetic resonance imaging (MRI) and colonoscopic biopsy confirmed descending colon adenocarcinoma with liver metastases, clinically staged as cT3N2M1a according to the eighth edition of the American Joint Committee on Cancer staging system (13). Immunohistochemical analysis showed retained mismatch repair protein expression (MLH1+, MSH2+, MSH6+, PMS2+) and HER2 1 +. While awaiting the results of next-generation sequencing (NGS) performed on the pretreatment primary-tumor biopsy using a 1,021-gene targeted panel (Chengdu Huachuang Qide Medical Laboratory Co. LTD. Chengdu, China) (14), the patient received one cycle of the CAPOX regimen (oxaliplatin and capecitabine).

In July 2024, NGS revealed microsatellite stability (MSS), low tumor mutational burden (TMB-L; 8.64 muts/Mb), KRAS/NRAS/BRAF wild-type status, and MET amplification with an estimated gene copy number (GCN) of 18.4. From July to November 2024, the patient received seven cycles of FOLFOX (oxaliplatin, leucovorin, and fluorouracil) plus cetuximab as first-line treatment. The best overall response during first-line therapy was partial response (PR). However, follow-up evaluation in November 2024 demonstrated progressive disease (PD), with both an increased number and enlargement of liver lesions compared with the CT and MRI findings obtained in September 2024.

**Figure 2:**
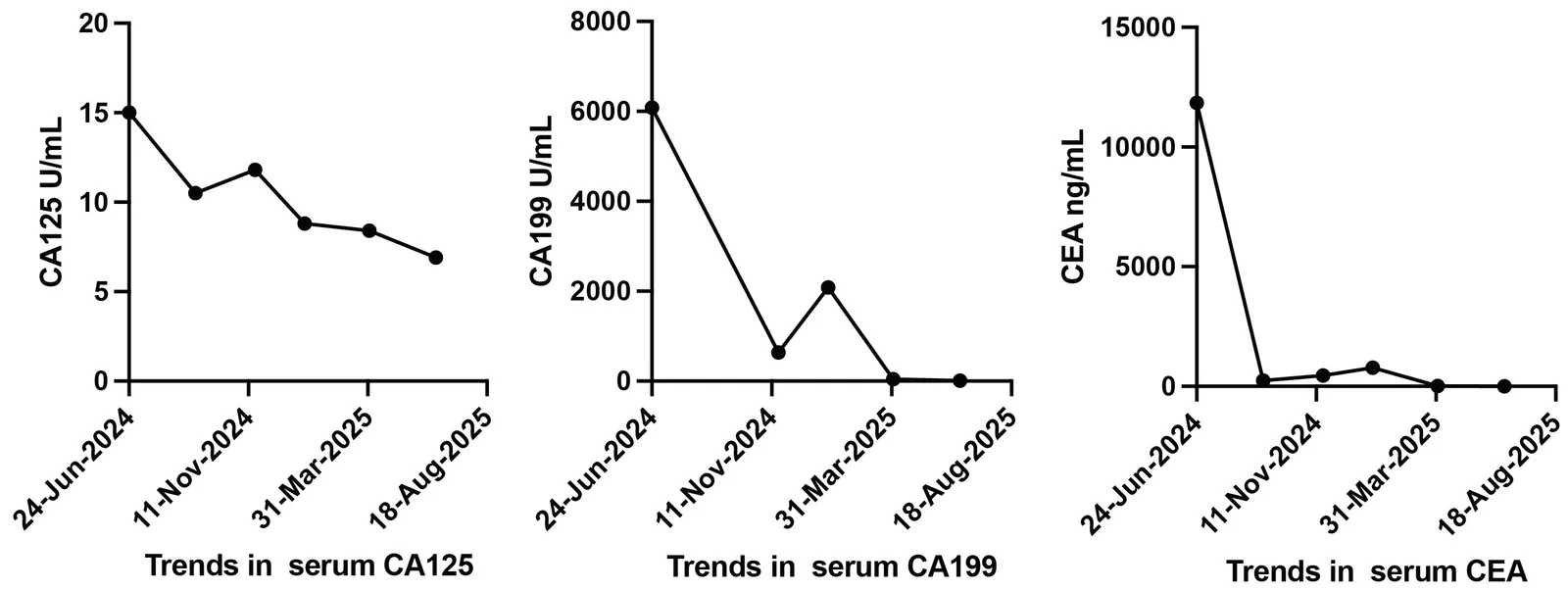
Dynamics of serum tumor markers during the entire disease course.

Accordingly, second-line treatment with FOLFIRI (irinotecan, leucovorin, and fluorouracil) plus bevacizumab was initiated on November 20, 2024. After three cycles, imaging evaluation in January 2025 showed stable disease (SD) (**Figure 1B**). However, serum tumor marker levels had increased compared with previous measurements (**Figure 2**). Although radiographic progression was not documented and stable disease indicated that FOLFIRI plus bevacizumab had achieved a degree of disease control, the concurrent increase in tumor-marker levels raised concern regarding the depth and durability of that control. Tumor-marker elevation alone was not considered evidence of disease progression. Given the presence of MET amplification, and based on prior evidence supporting the feasibility of combining MET-targeted therapy with chemotherapy and anti-EGFR or anti-VEGF agents in metastatic colorectal cancer, savolitinib was added to the ongoing second-line regimen in January 2025 (15, 16).

The patient received FOLFIRI plus bevacizumab during cycles 1–3. Savolitinib was introduced after cycle 3 in January 2025 at a dose of 300 mg orally once daily and was administered during cycles 4–11. Cycles 4–10 consisted of FOLFIRI plus bevacizumab and savolitinib. In preparation for surgery, bevacizumab was withheld during cycle 11, whereas dose-reduced FOLFIRI plus savolitinib was continued. Thus, the patient received a total of 11 cycles of preoperative FOLFIRI-based therapy. During combination treatment after the introduction of savolitinib, the patient developed grade 3 neutropenia and grade 1–2 nausea and vomiting. Owing to treatment-related toxicities, the FOLFIRI doses were reduced during cycles 8–11. The irinotecan dose was reduced from 280 to 240 mg on day 1, the fluorouracil bolus from 600 to 500 mg on day 1, and the 23-hour continuous fluorouracil infusion from 1,900 to 1,700 mg on each of days 1 and 2, administered every 2 weeks. During this period, treatment delay and/or dose adjustment were implemented in accordance with hematologic toxicity management principles. Serial radiologic assessments in April and June 2025 documented a partial response, with marked regression of the hepatic metastases accompanied by declining serum tumor-marker levels.

After 11 cycles of treatment, the patient underwent laparoscopic radical left hemicolectomy combined with resection of the left lateral hepatic lobe and liver metastases in July 2025. Pathological examination of the descending colon specimen showed moderately differentiated (grade 2) adenocarcinoma with residual tumor confined to the submucosa, corresponding to ypT1 according to the eighth edition of the AJCC staging system (13). Only single tumor cells or rare small groups of residual tumor cells remained, corresponding to tumor regression grade 1 according to the AJCC/College of American Pathologists modified Ryan grading system and indicating a near-complete pathological response (17). No lymphovascular or perineural invasion was identified, and all 15 examined pericolic lymph nodes were negative for metastasis (0/15), corresponding to ypN0. The primary tumor was therefore classified as ypT1N0. The resected hepatic lesions showed extensive treatment-related necrosis without identifiable viable tumor cells, indicating a pathological complete response.

The tissue-based baseline profiling component of a tumor-informed molecular residual disease (MRD) strategy was performed on the resected primary-tumor specimen in July 2025 using a 1,238-gene targeted NGS panel with matched peripheral blood leukocytes as the germline comparator. The estimated tumor-cell content was 10%, and the mean sequencing depth was 1,897×. No somatic alterations were detected, and MET amplification was not identified. Consequently, no patient-specific somatic variant could be selected for subsequent plasma ctDNA monitoring. Therefore, this tissue-based result should not be interpreted as postoperative MRD negativity or evidence of molecular remission.

Following surgery, the patient received four cycles of FOLFIRI plus bevacizumab and savolitinib, followed by two cycles of maintenance capecitabine, bevacizumab, and savolitinib. In February 2026, follow-up imaging showed newly developed hepatic abnormalities that were considered, after multidisciplinary review, to represent abnormal perfusion and focal fatty infiltration rather than recurrent metastatic disease. At the data cutoff in July 2026, approximately 12 months after surgery, no radiologically confirmed recurrence had been documented.

## Discussion

We report a rare case of a young woman with KRAS/NRAS/BRAF wild-type descending colon cancer and liver metastases with *de novo* MET amplification detected by NGS in a pretreatment primary-tumor biopsy. A profound radiologic and pathological response was observed during second-line treatment with continued FOLFIRI plus bevacizumab and added savolitinib, enabling conversion surgery. Nevertheless, this outcome represents the activity of the overall combination regimen, and the independent contribution of savolitinib cannot be established from a single uncontrolled case.

Mesenchymal-epithelial transition factor (MET) amplification is a rare but potentially actionable oncogenic alteration in metastatic colorectal cancer (mCRC) and has been implicated in tumor angiogenesis, invasion, metastasis, and therapeutic resistance (5–8, 18). However, studies of MET-amplified solid tumors have shown that the efficacy of MET inhibitor monotherapy is generally limited, particularly in mCRC, in which the best response reported in most studies has been stable disease (SD) (19–29). Clinical responses to MET-directed therapy may depend on the level, clonality, and biological context of MET amplification; however, standardized cross-platform criteria for defining MET amplification remain lacking (9, 30, 31). Nevertheless, compared with tumors harboring MET exon 14 skipping alterations, MET-amplified tumors tend to develop resistance to MET-targeted therapy earlier.

Several factors may account for the limited efficacy of MET inhibitor monotherapy. First, MET amplification is frequently accompanied by extensive crosstalk with other signaling pathways, particularly EGFR- and VEGF-related pathways. Consequently, MET inhibition alone may induce compensatory activation of downstream signaling cascades, including PI3K/AKT, MEK/MAPK, and hypoxia-related pathways, thereby attenuating antitumor activity (6, 32). Second, MET alterations may exhibit spatial heterogeneity between primary and metastatic lesions, potentially contributing to heterogeneous treatment responses (33, 34). However, molecular heterogeneity between the primary and metastatic sites was not demonstrated in the present patient. Third, the lack of standardized criteria and cutoff values for MET amplification complicates patient selection and limits accurate assessment of therapeutic efficacy (9, 31). Collectively, these challenges highlight the potential importance of combination strategies for advanced solid tumors harboring MET amplification, especially mCRC.

Given the biologic crosstalk between MET and EGFR, as well as between MET and VEGF, multiple clinical studies have explored the efficacy of combining a MET inhibitor with either an EGFR inhibitor or a VEGF inhibitor. In advanced lung cancer, MET inhibition combined with EGFR-targeted therapy has shown encouraging efficacy (35–37). In mCRC, however, although combination therapy appears to perform better than MET inhibitor monotherapy, the overall therapeutic benefit remains limited. For example, the combination of capmatinib and cetuximab in MET-positive mCRC achieved a best response of SD (38). In contrast, clinical evidence for the combined inhibition of VEGF and MET in mCRC remains extremely limited. To date, the only relevant study in another tumor type, high-grade glioma, showed that capmatinib plus bevacizumab failed to significantly improve outcomes, and treatment response was not associated with MET amplification status (39).

In addition to its role in resistance to targeted therapy, MET amplification has also been implicated in chemotherapy resistance (5). Preclinical studies have suggested that MET inhibition may reverse MET-mediated chemoresistance (5). However, the efficacy of combining a MET inhibitor with chemotherapy alone has also been suboptimal. In HER2-negative, MET-positive advanced gastric cancer, the addition of onartuzumab to mFOLFOX6 failed to significantly improve efficacy; although the onartuzumab group showed numerically higher ORR and PFS, the differences did not reach statistical significance (40).

Based on these findings, several studies have further evaluated regimens combining a MET inhibitor, chemotherapy, and either an EGFR inhibitor or a VEGF inhibitor. In patients with KRAS wild-type mCRC, the addition of tivantinib to irinotecan plus cetuximab did not significantly improve PFS in the overall study population (15). Although patients with high MET expression showed a possible trend toward benefit, with modest improvements in ORR and PFS in the tivantinib group, these differences were not statistically significant (15). Similarly, in the GO27827 trial, the addition of onartuzumab to FOLFOX plus bevacizumab did not improve outcomes in mCRC, and no meaningful PFS benefit was observed even in the MET-positive subgroup (16). Overall, the available evidence suggests that combining MET inhibitors with EGFR- or VEGF-targeted therapy plus chemotherapy has thus far yielded disappointing efficacy in broadly defined gastrointestinal tumor populations.

Several factors may explain the limited efficacy of these combination strategies in gastrointestinal tumors, particularly mCRC. First, the interactions between MET signaling and other molecular pathways are highly context-dependent and may differ across tumor types. Second, most available studies have enrolled small and molecularly heterogeneous cohorts, whereas clinical data specifically focusing on primary MET-amplified mCRC remain extremely limited. Most published trials are phase I-II studies, and some were terminated early because of poor accrual (41). Third, the absence of standardized assays and thresholds for defining MET amplification remains a major obstacle (9, 31). In addition, differences among MET inhibitors may also contribute. MET-targeted agents are broadly classified into tyrosine kinase inhibitors (TKIs) and anti-c-MET monoclonal antibodies (41). In tumors harboring MET amplification, TKIs may be more effective than monoclonal antibodies, such as onartuzumab, because they directly inhibit the intracellular kinase domain (16, 42). MET TKIs are further categorized into different subtypes, including type I and type II inhibitors (41–43). Type I MET TKIs selectively bind the active conformation of MET; however, clinical evidence supporting combination regimens based on type I MET inhibitors in MET-amplified mCRC remains scarce.

Savolitinib is an oral, highly selective type Ib MET TKI that inhibits MET receptor activity through specific interaction with the Y1230 residue (44). Compared with earlier or less selective MET-targeted agents, savolitinib offers greater target selectivity and has demonstrated a relatively favorable safety profile in clinical practice. It has also accumulated a comparatively higher level of clinical evidence, including data from phase IIIb studies demonstrating clinical activity in selected MET-driven malignancies (12, 44). In mCRC, however, MET inhibitor monotherapy has generally produced no better than SD (21). With regard to combination therapy, a preclinical study showed that savolitinib combined with the anti-VEGFR agent apatinib exerted a synergistic antitumor effect in colorectal cancer patient-derived xenograft models, including a c-MET-amplified model, although corresponding clinical data remain unavailable (45). Historical studies reported objective response rates of approximately 26%–32% with second-line FOLFIRI plus bevacizumab (46, 47), and pathological complete regression of colorectal liver metastases has occasionally been observed with this regimen alone (48). Therefore, the response in this patient cannot be attributed exclusively to savolitinib. Nevertheless, the temporal association between savolitinib initiation and subsequent sustained tumor regression suggests a possible additive effect of MET inhibition, although this interpretation remains hypothesis-generating.

To our knowledge, clinical experience with savolitinib combined with FOLFIRI and bevacizumab in MET-amplified metastatic colorectal cancer has rarely been reported. In the present patient, the pretreatment primary-tumor biopsy was analyzed using a 1,021-gene targeted NGS panel, which identified MET amplification with an estimated gene copy number of 18.4 (14). The original laboratory report did not provide a separately validated cutoff for categorizing the alteration as “high-level.” Because definitions of MET amplification vary substantially among NGS platforms and are not directly interchangeable with FISH-based MET/CEP7 criteria, we describe the alteration as “MET amplification detected by NGS, with an estimated gene copy number of 18.4” (9, 14, 31, 49). Notably, after four months of first-line FOLFOX plus cetuximab, the patient developed progression of liver metastases. The progression-free survival (PFS) on first-line treatment was only 5 months, which was markedly shorter than the median PFS reported in phase III trials of chemotherapy plus cetuximab in left-sided, RAS wild-type mCRC (e.g., CRYSTAL, 12 months; FIRE-3, 10.7 months) (50). Although an initial PR was achieved, rapid progression of liver lesions followed, suggesting the possibility of MET-driven resistance, consistent with previous reports (5–8, 18).

During second-line treatment with FOLFIRI plus bevacizumab, the first radiologic assessment after three cycles showed stable disease. This finding indicated that the original regimen had already achieved a degree of disease control. However, the concurrent increase in serum tumor-marker levels raised concern regarding the depth and durability of that control. After the addition of savolitinib, partial response was documented on serial imaging in April and June 2025, with marked shrinkage of liver metastases and a decline in serum tumor marker levels. This response enabled successful conversion from unresectable to resectable disease. Postoperative pathology demonstrated complete regression of the hepatic lesions and tumor regression grade 1 (TRG 1) in the primary tumor. In addition, the tissue-based baseline profiling component of a tumor-informed MRD strategy did not identify somatic alterations suitable for subsequent plasma ctDNA tracking in the resected primary-tumor specimen. Consequently, postoperative MRD status could not be established. The profound pathological response observed in this patient is biologically compatible with preclinical evidence showing antitumor effects of MET suppression and combined MET/VEGF inhibition in colorectal cancer models (10, 45), although the specific contribution of savolitinib remains uncertain.

Taken together, this case demonstrates profound antitumor activity of the overall combination regimen of FOLFIRI, bevacizumab, and savolitinib in a patient with MET-amplified mCRC. The temporal relationship between savolitinib initiation and subsequent deeper tumor regression supports a possible additive contribution of MET inhibition; however, the continued administration of FOLFIRI plus bevacizumab precludes determination of the independent effect of savolitinib. Accordingly, this observation should be considered hypothesis-generating and requires validation in prospective, molecularly selected studies.

Several limitations should be considered when interpreting this case. First, the independent contribution of savolitinib cannot be determined because FOLFIRI and bevacizumab were continued after savolitinib initiation. Stable disease after the first three cycles indicated that the original second-line regimen had already achieved a degree of disease control. The subsequent response may therefore have resulted from the cumulative effects of chemotherapy and bevacizumab, an additional effect of savolitinib, or an interaction among these treatments.

Second, serum tumor-marker elevation was one of the factors considered when savolitinib was introduced. However, tumor-marker elevation alone does not establish radiographic progression or resistance to FOLFIRI plus bevacizumab.

Third, MET amplification was detected only in the pretreatment primary-tumor biopsy. Pretreatment liver-metastasis tissue was unavailable, and the resected hepatic lesions contained no viable tumor cells for postoperative genomic testing. Therefore, molecular concordance between the primary and metastatic lesions could not be established, and spatial heterogeneity remains untested rather than demonstrated in this patient.

Fourth, MET amplification was identified by NGS with an estimated gene copy number of 18.4, but the testing report did not provide a separately validated cutoff for categorizing amplification as “high-level.” Given the variability in amplification definitions across NGS and FISH-based assays, the copy-number result should be interpreted within the context of the assay used (9, 31).

Fifth, only the tissue-based baseline profiling component of a tumor-informed MRD strategy was completed. No somatic alterations suitable for subsequent plasma ctDNA tracking were identified; therefore, postoperative MRD status could not be established. The low residual tumor content may also have limited the sensitivity of tissue-based variant detection.

Sixth, the patient received four cycles of postoperative FOLFIRI plus bevacizumab and savolitinib, followed by two cycles of maintenance capecitabine, bevacizumab, and savolitinib. Therefore, subsequent disease control cannot be attributed solely to the preoperative regimen or surgery.

Seventh, this is a single-case report, and the findings cannot be generalized to the broader population of patients with MET-amplified mCRC.

Finally, although no radiologically confirmed recurrence had been documented at approximately 12 months after surgery, the follow-up period remains insufficient to determine long-term recurrence-free survival or overall survival. Longer surveillance is required.

## Conclusion

In mCRC harboring primary MET amplification, savolitinib combined with chemotherapy and bevacizumab may provide meaningful antitumor activity, with adverse events that appear manageable and tolerable. This case highlights the potential value of MET-directed combination therapy in a molecularly selected subgroup of mCRC and supports the need for further prospective studies to validate this strategy and to clarify the predictive relevance of the level and site of MET amplification.

## Data Availability

The raw data supporting the conclusions of this article will be made available by the authors, without undue reservation.
